# A cross - sectional investigation on the molecular infection rate and subtype distribution of *Blastocystis* among diarrhea patients in Zhuhai, Southern Guangdong Province, China

**DOI:** 10.3389/fcimb.2025.1732588

**Published:** 2025-12-17

**Authors:** Lijie Sun, Shudong Tan, Zhongkai Zhang, Guangxu Ren, Huicong Huang, Wei Zhao

**Affiliations:** 1 Department of Clinical Laboratory, The Fifth Affiliated Hospital Sun Yat-sen University, Zhuhai, China; 2 School of Basic Medical Sciences, Wenzhou Medical University, Wenzhou, China; 3 Department of Infection Office, The First Affiliated Hospital of University of Science and Technology of China (USTC), Division of Life Sciences and Medicine, University of Science and Technology of China, Hefei, Anhui, China

**Keywords:** *Blastocystis*, diarrheal patients, genetic, Guangdong, China, subtyping

## Abstract

**Introduction:**

*Blastocystis* sp. is a common zoonotic protozoan found in both humans and animals. Epidemiological investigations seldom concentrate on this parasite, frequently underestimating its medical significance. The current study determined the presence and genetic characteristics of *Blastocystis* sp. in diarrheal patients from Zhuhai City, China.

**Methods:**

A total of 283 fecal samples were collected from diarrheal patients for DNA extraction. *Blastocystis* subtypes (STs) were identified by analyzing the DNA sequences of the small ribosomal subunit of the parasite isolates.

**Results:**

The overall infection rate of *Blastocystis* among the patients was 4.9% (14/283). The infection rate of *Blastocystis* was significantly higher in female patients (8.1%) than in male patients (2.9%); it was also higher in the elderly (6.3%) and adults (6.7%) compared to children (2.9%), although the difference was not statistically significant. Additionally, the infection rates of *Blastocystis* in rural (5.0%) and urban (4.9%) areas were consistent. Three known STs, including ST1 (n = 5), ST2 (n = 1) and ST3 (n = 8) were identified. A total of nine sequences were obtained across the three STs of *Blastocystis*, including four known and five novel sequences.

**Conclusions:**

This study is the first to report on the distribution of *Blastocystis* STs among individuals with diarrhea in Zhuhai, China, revealing potential transmission of the parasite both from human-to-human and from animals to humans. Given the ongoing debate regarding the pathogenicity of *Blastocystis*, it is recommended that patients suffering from diarrhea be closely monitored to identify and address the underlying causes, thereby enhancing their health outcomes.

## 1 Introduction

*Blastocystis* is an intestinal parasite commonly found in humans and is estimated to have infected over one billion people worldwide (Kühnelová et al., 2025). Although the association between *Blastocystis* sp. and gastrointestinal disease is uncertain, infection with *Blastocystis* can cause severe illness, including diarrhea, in both immunocompromised and immunocompetent individuals (Pawelec-Pęciak et al., 2025; Popruk et al., 2021). Nonetheless, recent research indicates that *Blastocystis* is not a pathogen but rather a gut inhabitant that may even be beneficial (Deng and Tan, 2025). Evidence supports its links to improved cardiometabolic markers and suggests potential protective effects against intestinal inflammation (Piperni et al., 2024). Surveillance of diverse hosts and populations, particularly those displaying symptoms of diarrhea, offers critical insights into the pathogenesis of *Blastocystis* and its potential therapeutic applications. Meanwhile, research on *Blastocystis* ought to be specifically focused on genetic characterization, with the objective of discerning host specificity and promoting the discovery of potentially pathogenic variants.

PCR with Sanger sequencing has been extensively used for epidemiological surveillance; this method can identify subtypes of *Blastocystis* (Stensvold and Clark, 2016). To date, at least 44 subtypes have been acknowledged through sequence and phylogenetic analysis of the small ribosomal subunit RNA (*SSU* rRNA) (Santin et al., 2024). Although ST1 to ST4 represent the most prevalent subtypes detected in humans, ST5 to ST9, ST12 to ST14, ST16, ST35, and ST41 have also been identified within the human population (Hernández-Castro et al., 2023; Zhao et al., 2024). The extensive genetic diversity of *Blastocystis* may be the primary reason for the discrepancy in pathogenicity, as different lineages exhibit varied ecological roles within the host gut (Deng and Tan, 2025). Research findings have indicated that ST4 serves as a beneficial commensal microorganism, and its colonization can confer protection against colitis. In contrast, infection with ST7 might represent a potential risk factor for the onset of experimentally induced colitis (Deng et al., 2022, 2023). Therefore, conducting research on the subtype distribution of *Blastocystis* in symptomatic patients is of utmost significance for achieving an in - depth comprehension of its genetic variation and functional pathogenicity.

In China, the initial report of *Blastocystis* infection involved two children suffering from chronic diarrhea in Guangdong Province. Subsequently, over 12 provinces and municipalities in China reported cases of *Blastocystis* infection, primarily affecting children, diarrhea patients, immunocompromised individuals, and cancer patients (**Table 1**). Despite this data, the majority of studies conducted before 2019 utilized morphological or cultural methods, lacking molecular typing data (Deng et al., 2019). Consequently, our understanding of the pathogenicity and transmission patterns, as well as the routes of transmission, of *Blastocystis* is limited or insufficient.

**Table 1:** Prevalence and STs distribution of *Blastocystis* in humans from different provinces of China.

Provinces	% (No. positive/no. sampled)	ST (s) (n)	References
Chongqing	15.2 (71/466)	ST3(18), ST7(11), ST6(32), ST1(40)	(Ning et al., 2020)
	10.6 (21/198)	ST6 (10), ST3 (8), ST1 (2), ST7 (1)	(Kang et al., 2019)
Guangxi	6.0 (17/285)	ST3 ( 8), ST1 ( 6), ST6 (2), ST7 (1)	(Xu et al., 2021)
	43.3 (215/497)	ST1 (25), ST6 (1), ST1+ST6 (1), Unknown (78)	^a^(He et al., 2013)
Guizhou	7.9 (43/548)	ST3 (24), ST1 (11), ST7 (3), ST5 (2), ST2 (1), ST4 (1), ST15 (1)	(Fu et al., 2025)
Hainan	7.3 (144/1973)	ST3(87), ST1 (40), ST7 (15), ST6 (1), ST2 (1)	(Wang et al., 2024)
Heilongjiang	8.1 (31/384)	ST1 (3); ST3 (27); ST14 (1)	(Zhao et al., 2022)
	10.5 (6/57)	ST1 (2), ST2 (1), ST3 (3)	(Chen et al., 2023)
	2.4 (3/126)	ST5 (3)	(Zhu et al., 2020)
	7.1 (27/381)	ST1 (12), ST3 (15)	(Zhang et al., 2017)
Henan	11.0 (87/793)	ST1 (14), ST3 (55), ST4 (1), ST6(2), ST7 (15)	(Mei et al., 2023)
	3.1 (33/1070)	ST1 (7), ST3 (23), ST7 (3)	(Li et al., 2021)
Hebei	34.9 (390/1118)	ST2 (389), ST5 (1)	(Ma et al., 2020)
Shanghai	1.9 (29/1505)	ST3 (17), ST1 (6), ST2 (1), ST6 (1), ST1+ ST3 (2), Unknown (2)	(Li et al., 2007a)
Xinjiang	14.3 (87/609)	ST1 (38), ST2 (8), ST3 (41)	(Qi et al., 2020)
Yunan	9.5 (48/507)	ST3 (24), ST1 (16), ST4 (7), ST2 (1)	(Deng et al., 2020)
	4.5 (13/289)	ST1 (3), ST3 (8),ST4 (1), Unknown (1)	(Gong et al., 2019)
	32.6 (78/239)	ST3 (56), ST1 (16), ST2 (1), ST4 (1), ST1+ST2 (1), ST1+ST3 (1), Unknown (3)	(Li et al., 2007a)
	5.8 (10/170)	ST3 (6), ST1 (3), ST2 (1)	(Li et al., 2007b)
	3.7 (12/324)	ST1 (3), ST3 (2), ST4 (3), ST7 (3), ST12 (1)	(Teng et al., 2018)
	4.0 (58/1440)	ST1 (457), ST2 (1)	(Zhang et al., 2016)
Zhejiang	6.5 (67/1032)	ST1 (12), ST2 (5), ST3 (35), ST4 (12), ST7 (3)	(Zhao et al., 2024)
	2.0 (10/489)	ST1 (3), ST2 (5), ST3 (5)	(Liu et al., 2023)
	23.7 (153/646)	ST3 (93), ST1 (38), ST2 (7), ST4 (1), ST1+ST3 (6), ST1+ST2 (1), ST2+ST3 (1), Unknown (6)	(Li et al., 2007a)

a The study in Gangxi (He et al., 2013) showed discrepancies between the number of positive samples and ST counts. This discrepancy arose because sequencing analysis was not performed on all positive samples, but only on 105 samples.

Unknown: The ST is indeterminable.

**Figure 1:**
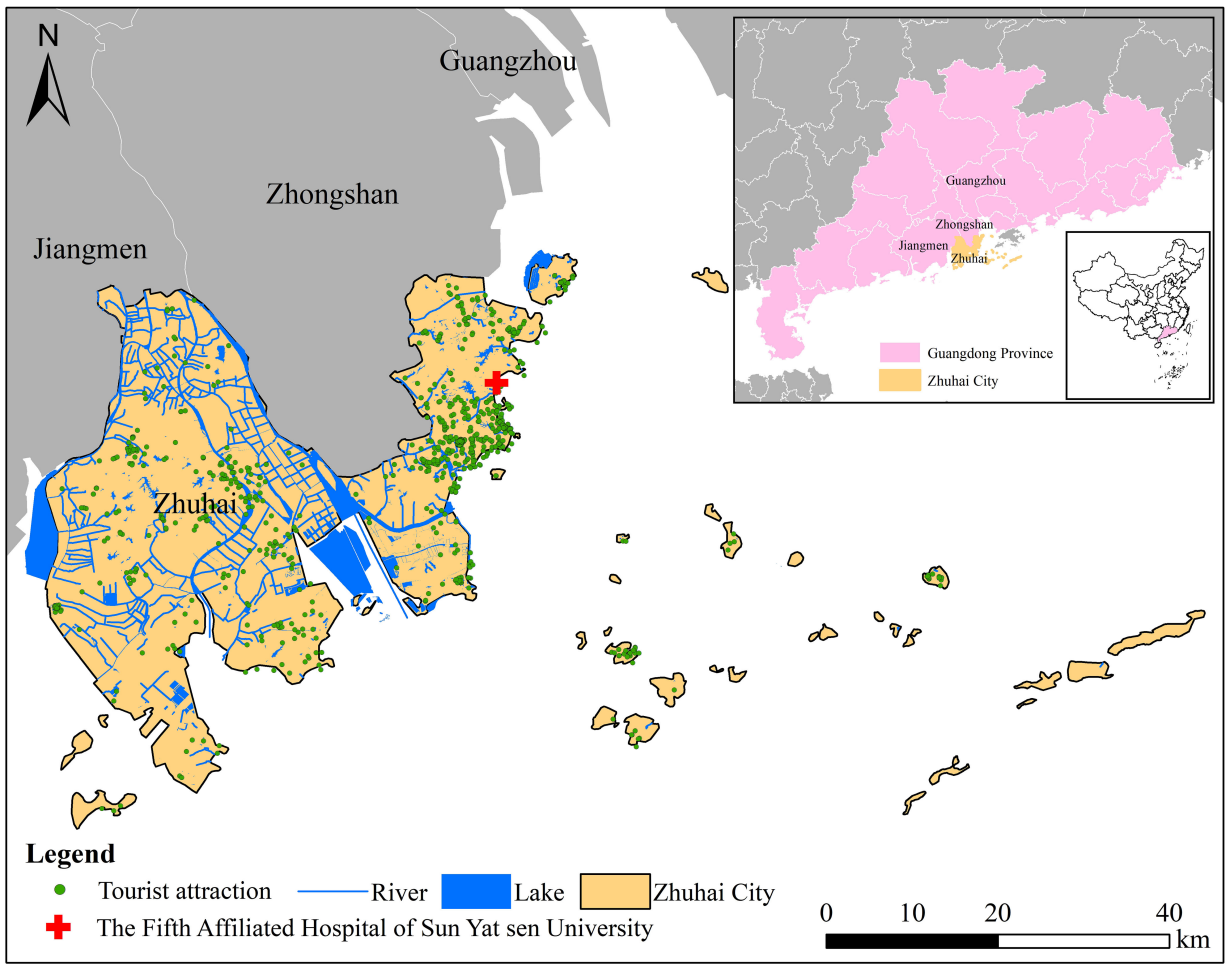
A map of Zhuhai City, Guangdong Province, China. The map includes information on tourist attractions, the distribution of rivers and lakes, and the sampling locations for this study. The researchers initially conceptualized and created the map using ArcGIS 10.4 software. The original vector diagram was imported from the National Geomatics Center of China (http://www.ngcc.cn). However, to comply with specific attribution and permission requirements, the final version of the map was revised and compiled by integrating Microsoft PowerPoint 2003 and Adobe Photoshop CS6.

Zhuhai, a city in Guangdong Province, China (**Figure 1**), is distinguished by a dense network of rivers and lakes. As the city with the largest marine area, the greatest number of islands, and the longest coastline in the Pearl River Delta region, it boasts a thriving tourism industry and high population mobility. The warm and humid climate further facilitates the dissemination of intestinal protozoa. However, molecular data on *Blastocystis* infection in the Zhuhai population are still lacking. Therefore, this study employs genotype typing technology to detect *Blastocystis* in diarrhea patients in Zhuhai, with the aim of comprehending the prevalence and genetic subtypes of *Blastocystis* in the region. This provides fundamental data for the in - depth exploration of *Blastocystis*’s epidemiology and prevention, as well as its pathogenic mechanisms and their association with public health.

## Materials and methods

### Ethics statement

The protocol of this study was approved by the Research Ethics Committee of Wenzhou Medical University (Approval number SCILLSC-2021-01, approved on April 3, 2020). Before sample collection, written informed consent was obtained from each participant or their legal guardians if the participant was a minor. Participants were fully informed about the study’s purpose, procedures, potential risks, and benefits, and were ensured that their participation was voluntary. Their personal information was kept confidential and used only for this research. The anonymity and privacy of the participants were strictly protected during the study.

### Specimen collection

From March 2023 to June 2024, a total of 283 fecal specimens were collected from diarrheal patients at the Laboratory Departments of the Fifth Affiliated Hospital of Sun Yat - sen University, situated in Zhuhai City, Guangdong Province, China (**Figure 1**, **Table 2**). The specimens were sourced from patients with fecal excretion surpassing 200 mg and experiencing at least three episodes of diarrhea per day. Prior to sample collection, informed consent was procured from the patients or from the parents/guardians of minor patients. Subsequently, they were educated on the appropriate method for collecting fecal specimens with the utilization of a plastic fecal collector. All stool collection containers were labeled with the unique clinical record number (no patient names were included), along with the collection date. The collected specimens were subsequently refrigerated at 4°C and transported via cold chain within 24 hours to the laboratory at Wenzhou Medical University. There, they were aliquoted into three 1.5 mL microcentrifuge tubes and stored at -80°C until DNA extraction.

**Table 2:** Prevalence and STs distribution of *Blastocystis* in diarrheic patients from Zhuhai City of Guangdong Province, China.

Groups	No. positive/no. examined (%)	ST(s) (n)	*P-value*
Gender			*P* = 0.049
Males	5/172 (2.9)	ST3 (5)	
Females	9/111 (8.1)	ST1 (5); ST3 (3); ST2 (1)	
Ages			*P* = 0.383
Children	3/104 (2.9)	ST1 (1); ST3 (2)	
Adult	11/163 (6.7)	ST1 (4); ST2 (1); ST3 (5)	
Elderly	1/16 (6.3)	ST3 (1)	
Residential location			*P* = 0.964
Rural area	6/123 (4.9)	ST1 (2); ST2 (1); ST3 (3)	
Urban area	8/160 (5.0)	ST1 (3); ST3 (5)	
Total	14/283 (1.8)	ST1 (5); ST2 (1); ST3 (8)	

### Source of data

Clinicians retrieved information regarding patients’ age, gender, and location (rural or urban) from the patients’ medical records and documented it appropriately. Among the 283 patients, 104 were under 18 years of age, 163 were aged between 19 and 60 years, and 16 were 61 years old or above. Additionally, 172 were male and 111 were female. Of these patients, 123 were from rural areas and 160 were from urban areas. No other disease information was recorded or subjected to statistical analysis; however, it was verified that all these patients were HIV-negative.

### DNA extraction and PCR

Genomic DNA was extracted from 180–200 mg of fecal samples using a QIAamp DNA Stool Mini Kit (QIAgen, Hilden, Germany) according to the manufacturer’s -protocol. The DNA was eluted in 200 μL of AE buffer. The 500 bp nucleotide fragment of the *small subunit* (*SSU*) *rRNA* gene of *Blastocystis* was amplified using PCR. The PCR was performed as described by Santín et al. (2011). TaKaRa Taq DNA Polymerase (TaKaRa Bio Inc., Tokyo, Japan) was used in all tests with a negative control (without DNA) and a positive control (DNA of the *Blastocystis* ST5 from pig) (Zhao et al., 2025). The PCR products were separated in 1.5% agarose gel electrophoresis and visualized using a Gel Doc EZ UV-gel imaging system (Bio-Rad Inc., USA). The gel was stained with GelRed (Biotium Inc., Hayward, CA) to aid visualization.

### Nucleotide sequencing and analysis

The PCR products were sequenced by Sangon Biotech (Shanghai) Co., Ltd. The quality of the sequence was assessed using two-directional sequencing. PCR products for DNA samples with novel sequences were further sequenced. The sequences were cleaned and aligned using DNASTAR Lasergene EditSeq v7.1.0 (http://www.dnastar.com/) and Clustal X v2.1 (http://www.clustal.org/) tools. The STs of *Blastocystis* were identified by comparing the sequenced data with that in GenBank using the Basic Local Alignment Search Tool (BLAST) (http://blast.ncbi.nlm.nih.gov/Blast.cgi).

### Statistical analyses

The data were analyzed using SPSS software version 22.0 (SPSS Inc., Chicago, IL, USA). The disparity in the prevalence of *Blastocystis* among patients of varying ages, genders, and residences was analyzed via the chi-square test with a 95% confidence interval. Statistical significance was defined as *P* < 0.05.

### Nucleotide sequence accession numbers

The novel ST sequences of *Blastocystis* obtained in the present study were deposited in the GenBank, and the accession numbers were PX472048 to PX472056.

## Results

### Prevalence of Blastocystis in diarrheic patients

*Blastocystis* was detected in 14 out of the 283(4.9%) sampled diarrheic patients (**Table 2**). In terms of gender, *Blastocystis* was detected in 2.9% (5/172) of male patients and 8.1% (9/111) of female patients, and this disparity was statistically significant (χ^2^ = 3.881, df = 1, *P* = 0.049). Concerning age, *Blastocystis* was detected in 2.9% (3/104) of children, 6.7% (11/163) of adults, and 6.3% (1/16) of elderly individuals, and there was no significant difference (χ^2^ = 1.919, df = 2, *P* = 0.383). Regarding residence, the *Blastocystis infection* rate in rural areas was 4.9% (6/123), which was consistent with that in urban areas at 5.0% (8/160).

### Distribution of *Blastocystis*’ STs

All 14 isolated *Blastocystis* subtypes were successfully sequenced. Three subtypes (STs), namely ST1, ST2, and ST3, were identified. Among them, ST3 was the most prevalent subtype, accounting for 57.1% (8/14), followed by ST1 at 35.7% (5/14), and ST2 at 7.1% (1/14). Notably, ST3 was the only subtype detected in male patients, while female patients harbored ST1, ST2, and ST3. Specifically, ST1 and ST3 were detected in children, ST1, ST2, and ST3 were identified in adults, and ST3 was the sole subtype present in the elderly. Both rural and urban patients carried ST1 and ST3, and ST2 was also detected in rural patients (**Table 2**).

### Genetic diversity of *Blastocystis*’ STs

The eight ST3 sequences generated five representative sequences, among which a total of six nucleotide variation sites were detected (**Table 3**). ST3-1 (PX472048) (n = 3) is identical to the KF242060 sequence from a Dutch individual; ST3-2 (PX472049) (n = 2) shows 100% homology with the KX108724 sequence from a Malaysian individual; ST3-3 (PX472050) (n = 1) matches the KF242064 sequence identified in a Dutch individual. The remaining two ST3 sequences, ST3-4 (PX472051) (n = 1) and ST3-5 (PX472052) (n = 1), have not been previously reported and differ from the previously reported KF242060 sequence by only one base each.

**Table 3:** Variation at six polymorphic sites within the five representative sequences of ST3 isolates identified in this study.

GenBank accession no.	Reference no.	Nucleotide at position					
		180	182	185	241	279	344
PX472048	KF242060	A	G	T	A	T	G
PX472049	KX108724	T	C	C	C	T	C
PX472050	KF242064	A	G	C	T	T	G
PX472051	KF242060	A	G	C	A	T	G
PX472052	KF242060	A	G	T	A	A	G

Among the five ST1 isolates, three isolates had identical sequences (PX472053) and showed 100% similarity to the PQ314630 sequence, which was derived from a human in Hainan, China; the sequences produced by the other two isolates (PX472054 and PX472055) had not been described before, differing by one base from the MK874819 sequence from rural populations in Mexico. A total of 22 nucleotide variation sites were identified among the three ST1 representative sequences obtained in this study (**Table 4**). The ST2 sequence (PX472056) is also a newly identified sequence, differing by one base (at position 241, T to C) from the OP503626 sequence isolated from rabbits in Spain.

**Table 4:** Variation at 22 polymorphic sites within the three representative sequences of ST1 isolates identified in this study.

GenBank accession no.	Reference no.	Nucleotide at position																					
		186	188	189	191	192	193	199	200	207	208	212	214	215	216	217	218	219	210	221	222	223	280
PX472053	PQ314630	A	C	G	T	G	T	G	A	T	C	C	A	C	A	G	T	A	A	G	T	C	C
PX472054	MK874819	G	G	C	A	T	C	A	G	C	T	A	G	A	T	A	C	T	C	A	C	T	G
PX472055	MK874819	G	G	C	A	T	C	A	A	C	T	A	G	A	T	A	C	T	C	A	C	T	A

## Discussion

In the present study, the mean infection rate of *Blastocystis* in surveyed diarrhea patients was 4.9%, consistent with previously reported rates in diarrhea patients from Yunnan, China (4.8%) and in Qatari children with diarrhea (4.7%) (Zhang et al., 2016; Boughattas et al., 2017). However, it was lower than the rates in patients with diarrhea in Indonesia (5.7%), Korea (11.6%), Iran (67.1%), and Turkey (5.9%), and higher than in patients with diarrhea in Singapore (1.0%) and children with diarrhea in Nepal (0.9%) (Maçin et al., 2016; Kwon et al., 2024; Oyofo et al., 2002; Koltas et al., 2017; Dahal et al., 2017; Feurle et al., 2021). Of the 24 molecular survey studies conducted in China, only six reported infection rates in a specific region that were lower than those found in this study, while the other 18 studies indicated infection rates higher than those in this study (**Table 1**). Certainly, when comparing these datasets, it is essential to account for variations among the survey subjects, including those from HIV-positive populations, cancer patients, children, healthy individuals, and those with other medical conditions. For instance, our previous survey found that the prevalence rate of *Blastocystis* spp. in diarrheic children in Zhejiang (8.8%) was significantly higher than that in asymptomatic children (2.0%) (Zhao et al., 2024). Overall, the population in China exhibits a low prevalence of *Blastocystis*, which may be associated with drinking habits. Individuals in China frequently consume boiled water and seldom drink untreated water, potentially decreasing the likelihood of ingesting infectious-stage *Blastocystis* and thus lowering the infection rate. Previous studies have indicated that in a mountain village in Yunnan Province, China, *Blastocystis* infection was associated with drinking unboiled water (Deng et al., 2020). Meanwhile, outbreaks of human *Blastocystis* infection were observed in China, resulting in over 1,122 cases of diarrhea confirmed after consuming contaminated drinking water (Wu et al., 2000). Given the high prevalence of *Blastocystis* in China’s animal populations, its frequent detection in aquatic environments, and its presence on the surfaces of vegetables and fruits, the population remains at significant risk (Deng et al., 2019; Li et al., 2020). Continuous monitoring of *Blastocystis* prevalence across different demographics is essential to better understand its pathogenicity and its relationship with public health.

Similar to some opportunistic pathogens, *Blastocystis* prevalence may be influenced by factors like host immunity, age, occupation, and climate. Detection methods are crucial for detection rates. Molecular detection techniques are more sensitive and specific than morphological examination and culture methods (Tan, 2008). In North Cyprus, *Blastocystis* prevalence in humans was 10.5%, 10.5%, and 27.8% by direct microscopy, trichrome method, and PCR respectively (Seyer et al., 2017). In a study of school - aged children in Colombia, it was 25.19% by microscopy and 39.22% by PCR (Villamizar et al., 2019). However, molecular techniques such as PCR can be inhibited by various factors; for instance, PCR inhibition in fecal DNA samples can lead to false-negative results. Additionally, primer pairs may produce nonspecific amplicons, particularly when screening directly extracted fecal DNA (Clark et al., 2013). For example, PCR using low-specificity eukaryotic primers may preferentially amplify shorter and more abundant *SSU rDNA* from co-infecting or co-colonizing organisms present in the intestine. Therefore, it is necessary to develop standard epidemiological methods for detecting *Blastocystis* (Tan, 2008). It is recommended to use Real-Time PCR in investigations, as it not only screens samples for positivity but also assays to estimate *Blastocystis* infection intensity in healthy carriers and patients with gastrointestinal issues, which would clarify the potential link between parasite intensity and symptoms (Stensvold et al., 2012).

*Blastocystis* infection is commonly observed in children. For instance, Gong et al.’s survey on the population at the China-Myanmar border revealed that the prevalence of *Blastocystis* in children (12.37%) is significantly higher than in adults (4.95%) (Gong et al., 2019). However, contrary research results have also been described, indicating that the colonization of *Blastocystis* is linearly related to age, meaning that the infection rate of *Blastocystis* increases with age. It could be hypothesised that *Blastocystis* is dependent on a more mature (diverse) microbiota in order to be able to establish (Stensvold, 2025). The current study indicates that the infection rate among adults exceeds that among children, although the difference is not statistically significant. However, this research discovered that females have a significantly higher infection rate (8.1%) compared to males (2.9%). In numerous households, women predominantly care for pets, which increases their exposure to potentially pathogen-carrying animals. Additionally, they may be more engaged in household chores that expose them to infection sources, such as cleaning areas frequented by animals or handling animal waste. These activities may result in females being more susceptible to infections than males. At the same time, due to the relatively small sample size and the number of positive cases, a definitive conclusion regarding the correlation with age or gender cannot yet be drawn. Consequently, further research is required to elucidate the relationship between age, gender, and *Blastocystis* infection.

It is generally believed that the infection rate in rural populations is higher than in urban populations because the prevalence of *Blastocystis* may be closely related to living environment and sanitation conditions, and the sanitary environment in cities is undoubtedly superior to that in rural areas (Deng et al., 2020). However, in some parts of China, the sanitary environment in rural areas has been widely improved, which may directly affect the prevalence of some parasitic diseases. In any case, the detection of *Blastocystis* prevalence in the population with diarrhea in this study suggests that these patients should be taken seriously. On the one hand, these patients require improvement in their clinical symptoms; on the other hand, they may become sources of infection, leading to infections in other populations and animals, thereby facilitating the spread of *Blastocystis*.

The increasing utilization of PCR-based methods has, likewise, expanded the comprehension of the genetic diversity and transmission of *Blastocystis* spp. Over the past decade, research on *Blastocystis* spp. at the subtype level has conspicuously intensified (Stensvold, 2025). Among the 44 subtypes identified, ST1 to ST4 are commonly found in humans (Zhao et al., 2024). In the current study, three known STs (ST1, ST2, and ST3) were detected, with ST3 being the most prevalent, followed by ST1. This finding aligns with studies conducted in other parts of the world, where subtype ST3 accounts for at least 44% of all human cases, and ST1 is the second-most predominant, causing over 27% of human cases (Popruk et al., 2021). In the present study, the proportion of ST2 was relatively low, with only one patient testing positive for ST2. This subtype has a relatively high proportion in populations of African, American, and European countries, while it has a relatively small proportion in Asian populations (Popruk et al., 2021). In China, except for the Hebei region, where ST2 was reported to be in absolute dominance, the proportion of the ST2 subtype was small in other regions (Ma et al., 2020). For example, in Hainan Province, among 144 reported human cases, only one case was ST2, and ST2 was not found in Chongqing, Guangxi, Guizhou, etc (**Table 1**). This indicates that the transmission of *Blastocystis* subtypes may exhibit regional prevalence. Additionally, in China, an increasing number of rare ST subtypes of *Blastocystis* have been identified in humans. For instance, two studies in Chongqing reported a high proportion of ST6 and ST7, and a study in Heilongjiang showed that only ST5 was present in the population; ST14 was also identified in HIV - infected individuals from Heilongjiang (**Table 1**). This may suggest that the mode of human infection may be shifting from human - to - human transmission to animal - to - human transmission, or that the animal - to - human transmission mode is increasing.

In Guangdong Province, the study area, documented cases of *Blastocystis* infection have been reported in various animals, including black goats, chickens, pigs, farmed bamboo rats, and dogs (Yu et al., 2023; Liu et al., 2022; Song et al., 2021; Zou et al., 2021; Liao et al., 2020). Subtype analysis indicates that farm animals predominantly carry ST1 to ST4 subtypes, which are less likely to infect humans. Conversely, the ST3 subtype is predominant among companion animals like pet dogs (Liao et al., 2020). While there is no direct evidence indicating that human infection with *Blastocystis* comes from pet dogs, the discovery of the ST3 subtype in both humans and dogs within the same region underscores the potential for transmission between the two. Therefore, this study suggests that the prevalent *Blastocystis* in surveyed diarrhea patients may not only spread through human-to-human transmission but also through pet-to-human and human-to-pet transmission routes.

In fact, an increasing number of infection studies indicate that contaminated water serves as a significant source of *Blastocystis* infections (Attah et al., 2023). Previous research in Yunnan Province of China found that consuming raw aquatic plants is associated with ST1 infections, while drinking unboiled water correlates with ST3 infections (Li et al., 2007b). Although specific data on *Blastocystis* contamination in Guangdong Province remains unavailable, other studies have confirmed the presence of *Blastocystis* in tap water. For instance, *Blastocystis* ST3 has been detected in tap water in northern Thailand (Jinatham et al., 2022). Therefore, the next step involves conducting a detection of *Blastocystis* in the water bodies of Guangdong Province to better infer and assess the possible sources of human *Blastocystis* infection.

## Conclusions

This study presents a preliminary exploration of the prevalence and genetic characteristics of *Blastocystis* in patients with diarrhea in Zhuhai, Guangdong Province, China. The results show an average infection rate of 4.9% in the investigated population. The infection rate is significantly higher in females than males, but there are no significant differences among age groups and residential areas. The identification of zoonotic ST1, ST2, and ST3 suggests the potential role of diarrhea patients in transmitting *Blastocystis* to both humans and animals. Although there is a lack of local farm animal infection data for ST1, ST2, and ST3, the high prevalence of ST3 in pet dogs indicates potential cross-transmission between humans and pets. Meanwhile, these patients experiencing diarrhea should be given attention to identify and address the underlying causes of the disease, thereby improving their health.

## Data Availability

The datasets presented in this study can be found in online repositories. The names of the repository/repositories and accession number(s) can be found in the article/supplementary material.

## References

[B1] Attah A. O. Sanggari A. Li L. I. Nik Him N. A. I. I. Ismail A. H. Meor Termizi F. H. (2023). Blastocystis occurrence in water sources worldwide from 2005 to 2022: a review. Parasitol. Res. 122, 1–10. doi: 10.1007/s00436-022-07731-0, PMID: 36434314

[B2] Boughattas S. Behnke J. M. Al-Ansari K. Sharma A. Abu-Alainin W. Al-Thani A. . (2017). Molecular analysis of the enteric protozoa associated with acute diarrhea in hospitalized children. Front. Cell. Infect. Microbiol. 7, 343. doi: 10.3389/fcimb.2017.00343, PMID: 28824878 PMC5539595

[B3] Chen H. Hao Y. Liu Y. Xu M. Zhang W. Li H. . (2023). The frequency and subtype distribution of *Blastocystis* sp. in humans and domestic animals in households in Heilongjiang Province, China. Acta Trop. 240, 106844. doi: 10.1016/j.actatropica.2023.106844, PMID: 36706827

[B4] Clark C. G. van der Giezen M. Alfellani M. A. Stensvold C. R. (2013). Recent developments in *Blastocystis* research. Adv. Parasitol. 82, 1–32. doi: 10.1016/B978-0-12-407706-5.00001-0, PMID: 23548084

[B5] Dahal M. Dahal R. H. Chaudhary D. K. (2017). Prevalence of cyclospora cayetanensis and other enteropathogen among childrenunder the age of 15 years in Biratnagar, Nepal. Asian Pac. J. Trop. Dis. 7, 75–79. doi: 10.12980/apjtd.7.2017D6-342

[B6] Deng L. Chai Y. Zhou Z. Liu H. Zhong Z. Hu Y. . (2019). Epidemiology of *Blastocystis* sp. infection in China: a systematic review. Parasite 26, 41. doi: 10.1051/parasite/2019042, PMID: 31309925 PMC6632114

[B7] Deng L. Tan K. S. W. (2025). From parasite to partner: unravelling the multifaceted role of *Blastocystis* in human health and disease. Lancet Microbe. 6, 101155. doi: 10.1016/j.lanmic.2025.101155, PMID: 40541220

[B8] Deng L. Wojciech L. Png C. W. Kioh D. Y. Q. Gu Y. Aung T. T. . (2023). Colonization with two different *Blastocystis* subtypes in DSS-induced colitis mice is associated with strikingly different microbiome and pathological features. Theranostics. 13, 1165–1179. doi: 10.7150/thno.81583, PMID: 36793854 PMC9925320

[B9] Deng L. Wojciech L. Png C. W. Koh E. Y. Aung T. T. Kioh D. Y. Q. . (2022). Experimental colonization with *Blastocystis* ST4 is associated with protective immune responses and modulation of gut microbiome in a DSS-induced colitis mouse model. Cell Mol. Life Sci. 79, 245. doi: 10.1007/s00018-022-04271-9, PMID: 35435504 PMC9016058

[B10] Deng Y. Zhang S. Ning C. Zhou Y. Teng X. Wu X. . (2020). Molecular epidemiology and risk factors of Blastocystis sp. Infections among general populations in Yunnan province, Southwestern China. Risk Manag Healthc Policy. 13, 1791–1801. doi: 10.2147/RMHP.S269664, PMID: 33061712 PMC7532910

[B11] Feurle G. E. Moos V. Landt O. Corcoran C. Reischl U. Maiwald M. (2021). Tropheryma whipplei in feces of patients with diarrheain 3 locations on different continents. Emerg. Infect. Dis. 27, 932–935. doi: 10.3201/eid2703.200182, PMID: 33622479 PMC7920677

[B12] Fu X. Lyu J. Shi Y. Cao B. Liu D. Yang X. . (2025). Epidemiological survey on prevalence and subtypes distribution of *Blastocystis* sp. in Southern Guizhou, China. Biomol Biomed. 25, 1508–1516. doi: 10.17305/bb.2024.11303, PMID: 39636276 PMC12097392

[B13] Gong B. Liu X. Wu Y. Xu N. Xu M. Yang F. . (2019). Prevalence and subtype distribution of *Blastocystis* in ethnic minority groups on both sides of the China-Myanmar border, and assessment of risk factors. Parasite. 26, 46. doi: 10.1051/parasite/2019046, PMID: 31343971 PMC6658150

[B14] He S. S. Wu L. Y. Liu X. Q. Shi H. H. Chen Z. Zhang H. . (2013). Investigation on the infection of *Blastocystis hominis* in populations in Bama Yao Autonomous County of Guangxi. Zhongguo Ji Sheng Chong Xue Yu Ji Sheng Chong Bing Za Zhi. 31, 76–77., PMID: 24812846

[B15] Hernández-Castro C. Maloney J. G. Agudelo-López S. P. Toro-Londoño M. A. Botero-Garcés J. H. Orozco M. C. . (2023). Identification and validation of novel *Blastocystis* subtype ST41 in a Colombian patient undergoing colorectal cancer screening. J. Eukaryot Microbiol. 70, e12978. doi: 10.1111/jeu.12978, PMID: 37195413

[B16] Jinatham V. Nonebudsri C. Wandee T. Popluechai S. Tsaousis A. D. Gentekaki E. (2022). *Blastocystis* in tap water of a community in northern Thailand. Parasitol. Int. 91, 102624. doi: 10.1016/j.parint.2022.102624, PMID: 35842087

[B17] Kang J. M. Li Y. T. Chen R. Yu Y. F. Li X. T. Wu X. P. . (2019). Prevalence and risk factors of Blastocystis hominis infection in inpatients in Jiangjin District, Chongqing City. Zhongguo Xue Xi Chong Bing Fang Zhi Za Zhi. 31, 479–485., PMID: 31713375 10.16250/j.32.1374.2018244

[B18] Koltas I. S. Elgun G. Eroglu F. Demirkazık M. (2017). The importance of real-time polymerase chain reaction method in diagnosisof intestinal parasites in cases with diarrhea. Trop. Biomed. 34, 895–902., PMID: 33592959

[B19] Kühnelová S. Rožnovský L. Doležílková J. Maďar R. (2025). Blastocystis in the human gastrointestinal tract - commensal or “silent” pathogen? Epidemiol. Mikrobiol Imunol. 74, 118–125. doi: 10.61568/emi/11-6492/20250428/140419, PMID: 40747753

[B20] Kwon J. Y. Choi J. H. Lee H. I. Ju J. W. Lee M. R. (2024). Molecular prevalence of *Blastocystis* sp. from patients with diarrhea in the Republic of Korea. Microorganisms. 12, 523. doi: 10.3390/microorganisms12030523, PMID: 38543574 PMC10972355

[B21] Li J. Dong H. Karim M. R. Yang X. Chao L. Liu S. . (2021). Molecular identification and subtyping of *Blastocystis* sp. in hospital patients in Central China. Eur. J. Protistol. 79, 125796. doi: 10.1016/j.ejop.2021.125796, PMID: 33975057

[B22] Li J. Wang Z. Karim M. R. Zhang L. (2020). Detection of human intestinal protozoan parasites in vegetables and fruits: a review. Parasit Vectors. 13, 380. doi: 10.1186/s13071-020-04255-3, PMID: 32727529 PMC7392835

[B23] Li L. H. Zhang X. P. Lv S. Zhang L. Yoshikawa H. Wu Z. . (2007a). Cross-sectional surveys and subtype classification of human Blastocystis isolates from four epidemiological settings in China. Parasitol. Res. 102, 83–90. doi: 10.1007/s00436-007-0727-0, PMID: 17912552

[B24] Li L. H. Zhou X. N. Du Z. W. Wang X. Z. Wang L. B. Jiang J. Y. . (2007b). Molecular epidemiology of human Blastocystis in a village in Yunnan province, China. Parasitol. Int. 56, 281–286. doi: 10.1016/j.parint.2007.06.001, PMID: 17627869

[B25] Liao S. Lin X. Sun Y. Qi N. Lv M. Wu C. . (2020). Occurrence and genotypes of *Cryptosporidium* spp., *Giardia duodenalis*, and *Blastocystis* sp. in household, shelter, breeding, and pet market dogs in Guangzhou, southern China. Sci. Rep. 10, 17736. doi: 10.1038/s41598-020-74299-z, PMID: 33082394 PMC7576217

[B26] Liu X. Ni F. Li J. Wang R. Yang X. Ge Y. . (2022). Research Note: Prevalence and zoonotic concern of *Blastocystis* in farmed chickens in southern China. Poult Sci. 101, 102182. doi: 10.1016/j.psj.2022.102182, PMID: 36228529 PMC9573914

[B27] Liu H. Ni H. Zhu N. Liu S. Wang R. Cao J. . (2023). *Blastocystis* infection among diarrhea outpatients in Ningbo, Southeast China: A potential zoonotic health threat. Microb. Pathog. 181, 106219. doi: 10.1016/j.micpath.2023.106219, PMID: 37391101

[B28] Ma L. Qiao H. Wang H. Li S. Zhai P. Huang J. . (2020). Molecular prevalence and subtypes of *Blastocystis* sp. in primates in northern China. Transbound Emerg. Dis. 67, 2789–2796. doi: 10.1111/tbed.13644, PMID: 32445593

[B29] Maçin S. Kaya F. Çağdaş D. Hizarcioglu-Gulsen H. Saltik-Temizel I. N. Tezcan İ. . (2016). Detectionof parasites in children with chronic diarrhea. Pediatr. Int. 58, 531–533. doi: 10.1111/ped.12959, PMID: 27322863

[B30] Mei X. Su C. Wang W. Zhang B. Wei L. Zhang Z. . (2023). Molecular prevalence and subtypes distribution of *Blastocystis* sp. among outpatients and inpatients in north and south areas of Henan Province, China. J. Eukaryot Microbiol. 70, e12960. doi: 10.1111/jeu.12960, PMID: 36478629

[B31] Ning C. Q. Kang J. M. Li Y. T. Chen H. H. Chu Y. H. Yu Y. F. . (2020). Prevalence and risk factors of Blastocystis infections among primary school students in Jiangjin District, Chongqing City. Zhongguo Xue Xi Chong Bing Fang Zhi Za Zhi. 32, 489–497., PMID: 33185060 10.16250/j.32.1374.2020189

[B32] Oyofo B. A. Subekti D. Tjaniadi P. Machpud N. Komalarini S. Setiawan B. . (2002). Enteropathogens associated with acute diarrhea in community and hospital patients in Jakarta, Indonesia. FEMS Immunol. Med. Microbiol. 34, 139–146. doi: 10.1111/j.1574-695X.2002.tb00615.x, PMID: 12381465

[B33] Pawelec-Pęciak O. Łanocha-Arendarczyk N. Grzeszczak K. Kosik-Bogacka D. (2025). The role of *Blastocystis* spp. in the etiology of gastrointestinal and autoimmune diseases. Pathogens. 14, 313. doi: 10.3390/pathogens14040313, PMID: 40333047 PMC12030515

[B34] Piperni E. Nguyen L. H. Manghi P. Kim H. Pasolli E. Andreu-Sánchez S. . (2024). Intestinal *Blastocystis* is linked to healthier diets and more favorable cardiometabolic outcomes in 56,989 individuals from 32 countries. Cell. 187, 4554–4570.e18. doi: 10.1016/j.cell.2024.06.018, PMID: 38981480

[B35] Popruk S. Adao D. E. V. Rivera W. L. (2021). Epidemiology and subtype distribution of *Blastocystis* in humans: A review. Infect. Genet. Evol. 95, 105085. doi: 10.1016/j.meegid.2021.105085, PMID: 34530156

[B36] Qi M. Wei Z. Zhang Y. Zhang Q. Li J. Zhang L. . (2020). Genetic diversity of *Blastocystis* in kindergarten children in southern Xinjiang, China. Parasit Vectors. 13, 15. doi: 10.1186/s13071-020-3890-0, PMID: 31924261 PMC6954523

[B37] Santin M. Figueiredo A. Molokin A. George N. S. Köster P. C. Dashti A. . (2024). Division of *Blastocystis* ST10 into three new subtypes: ST42-ST44. J. Eukaryot Microbiol. 71, e12998. doi: 10.1111/jeu.12998, PMID: 37658622

[B38] Santín M. Gómez-Muñoz M. T. Solano-Aguilar G. Fayer R. (2011). Development of a new PCR protocol to detect and subtype *Blastocystis* spp. from humans and animals. Parasitol. Res. 109, 205–212. doi: 10.1007/s00436-010-2244-9, PMID: 21210149

[B39] Seyer A. Karasartova D. Ruh E. Güreser A. S. Turgal E. Imir T. . (2017). Epidemiology and prevalence of *Blastocystis* spp. in north Cyprus. Am. J. Trop. Med. Hyg. 96, 1164–1170. doi: 10.4269/ajtmh.16-0706, PMID: 28167596 PMC5417212

[B40] Song J. Yang X. Ma X. Wu X. Wang Y. Li Z. . (2021). Molecular characterization of *Blastocystis* sp. in Chinese bamboo rats (*Rhizomys sinensis*). Parasite. 28, 81. doi: 10.1051/parasite/2021081, PMID: 34907896 PMC8672676

[B41] Stensvold C. R. (2025). Aspects of genetic diversity, host specificity and public health significance of single-celled intestinal parasites commonly observed in humans and mostly referred to as ‘Non-pathogenic’. APMIS 133, e70036. doi: 10.1111/apm.70036, PMID: 40923351 PMC12418315

[B42] Stensvold C. R. Ahmed U. N. Andersen L. O. Nielsen H. V. (2012). Development and evaluation of a genus-specific, probe-based, internal-process-controlled real-time PCR assay for sensitive and specific detection of *Blastocystis* spp. J. Clin. Microbiol. 50, 1847–1851. doi: 10.1128/JCM.00007-12, PMID: 22422846 PMC3372105

[B43] Stensvold C. R. Clark C. G. (2016). Current status of *Blastocystis*: A personal view. Parasitol. Int. 65, 763–771. doi: 10.1016/j.parint.2016.05.015, PMID: 27247124

[B44] Tan K. S. (2008). New insights on classification, identification, and clinical relevance of *Blastocystis* spp. Clin. Microbiol. Rev. 21, 639–665. doi: 10.1128/CMR.00022-08, PMID: 18854485 PMC2570156

[B45] Teng X. Chu Y. Zhai C. Yu Y. Cai Y. Chen S. . (2018). The epidemio−logical characteristics and infuencing factors for Blastocystis hominis infection among human immunodefciency virus seropositive individuals in Tengchong of Yunnan Province. Zhong guo Ji Sheng Chong Xue Yu Ji Sheng Chong Bing Za Zhi 36, 129–134.

[B46] Villamizar X. Higuera A. Herrera G. Vasquez-A L. R. Buitron L. Muñoz L. M. . (2019). Molecular and descriptive epidemiology of intestinal protozoan parasites of children and their pets in Cauca, Colombia: a cross-sectional study. BMC Infect. Dis. 19, 190. doi: 10.1186/s12879-019-3810-0, PMID: 30808303 PMC6390308

[B47] Wang Y. Lai X. Liu R. Li J. Ren G. Lu X. . (2024). Molecular prevalence and subtype characteristics of *Blastocystis* among school children in Hainan, the tropical island province of China. Acta Trop. 258, 107353. doi: 10.1016/j.actatropica.2024.107353, PMID: 39122102

[B48] Wu G. Xiong Y. Cao G. Guang L. I. Liu M. Zhu J. (2000). Investigation of an epidimicoutbreak of blastocystisasis. Chin. J. Parasit Dis. Control. 13, 25–27.

[B49] Xu N. Jiang Z. Liu H. Jiang Y. Wang Z. Zhou D. . (2021). Prevalence and genetic characteristics of Blastocystis hominis and Cystoisospora belli in HIV/AIDS patients in Guangxi Zhuang Autonomous Region, China. Sci. Rep. 11, 15904. doi: 10.1038/s41598-021-94962-3, PMID: 34354101 PMC8342556

[B50] Yu X. Wang H. Li Y. Mu X. Yuan K. Wu A. . (2023). Occurrence and Genotypic Identification of *Blastocystis* spp., *Enterocytozoon bieneusi*, and *Giardia duodenalis* in Leizhou Black Goats in Zhanjiang City, Guangdong Province, China. Anim. (Basel) 13, 2777. doi: 10.3390/ani13172777, PMID: 37685041 PMC10486513

[B51] Zhang W. Ren G. Zhao W. Yang Z. Shen Y. Sun Y. . (2017). Genotyping of *Enterocytozoon bieneusi* and subtyping of *Blastocystis* in cancer patients: relationship to diarrhea and assessment of zoonotic transmission. Front. Microbiol. 8, 1835. doi: 10.3389/fmicb.2017.01835, PMID: 28983297 PMC5613175

[B52] Zhang S. X. Tian L. G. Lu Y. Li L. H. Chen J. X. Zhou X. N. (2016). Epidemiological characteristics of Blastocystis hominis in urban region, southwestern China. Zhong guo Ren Shou Gong Huan Bing Za Zhi 32, 424–428.

[B53] Zhao W. Ren G. Wang L. Xie L. Wang J. Mao J. . (2024). Molecular prevalence and subtype distribution of *Blastocystis* spp. among children who have diarrheia or are asymptomatic in Wenzhou, Zhejiang Province, China. Parasite. 31, 12. doi: 10.1051/parasite/2024012, PMID: 38450718 PMC10918642

[B54] Zhao W. Sun L. Sun Y. Fu X. Ma S. Zhang J. . (2025). Molecular identification and genotyping of *Blastocystis* in farmed Cattle, Goats, and Pigs from Zhejiang Province, China. Food waterborne Parasitol. 40, e00280. doi: 10.1016/j.fawpar.2025.e00280, PMID: 40809407 PMC12347955

[B55] Zhao W. Yao L. Zhuang M. Lin Y. L. Chen X. H. Wang L. . (2022). A baseline epidemiological study of the co-infection of enteric protozoans with human immunodeficiency virus among men who have sex with men from Northeast China. PloS Negl. Trop. Dis. 16, e0010712. doi: 10.1371/journal.pntd.0010712, PMID: 36067140 PMC9447920

[B56] Zhu W. Wei Z. Li Q. Lin Y. Yang H. Li W. (2020). Prevalence and subtype diversity of *Blastocystis* in human and nonhuman primates in North China. Parasitol. Res. 119, 2719–2725. doi: 10.1007/s00436-020-06761-w, PMID: 32524268

[B57] Zou Y. Yang W. B. Zou F. C. Lin R. Q. Zhu X. Q. Hou J. L. (2021). Molecular detection and subtype distribution of *Blastocystis* in farmed pigs in southern China. Microb. Pathog. 151, 104751. doi: 10.1016/j.micpath.2021.104751, PMID: 33482261

